# Compliance with Health-Related Behaviors Guidelines and Its Relationship with Multiple Factors in Preschool Children Aged 3–6 Years: A National Cross-Sectional Survey in China

**DOI:** 10.3390/ijerph19031262

**Published:** 2022-01-23

**Authors:** Weizhen Gao, Yanfeng Zhang, Dongming Wu, Yanhui Dong, Na Liu, Huan Wang

**Affiliations:** 1 National Fitness Survey Center, China Institute of Sport Science, Beijing 100061, China; gaoweizhen92@163.com (W.G.); zhangyanfeng@ciss.cn (Y.Z.); wudongming@ciss.cn (D.W.); 2 Institute of Child and Adolescent Health, School of Public Health, Peking University, Beijing 100191, China; dongyanhui@bjmu.edu.cn; 3 The Fifth Kindergarten of Chinese Academy of Sciences, Beijing 100101, China; m13625059579@163.com

**Keywords:** preschool children, moderate to vigorous physical activity, screen time behavior, sleep behavior, supportive family environmental factors, parental behavior factors

## Abstract

Purpose: To investigate the compliance rates of health-related behaviors among Chinese preschool children, and to explore how supportive family environment, parental behavior, sociodemographic and community factors affect children’s health-related behavior comprehensively. Method: Preschool children aged 3 to 6 years were chosen from 5760 villages (residential) committees from 471 counties (districts) of 31 provinces by use of a stratified random sampling procedure, with 10,967 preschool children aged 3–6 years old included. The survey was conducted from September 2020 to November 2020. Results: The proportion of Chinese preschool children who met the moderate to vigorous physical activity (MVPA), screen time behavior (ST), and sleep behavior (SLP) guidelines were 62.3%, 52.8%, and 53.8%. Among the supportive family environment factors, parents’ time with their children on weekends had the most significant impact on children’s MVPA, ST, and SLP, with the odds ratio (OR) values of 2.18 (95%CI:1.97, 2.40), 0.69 (0.63, 0.76), and 1.62 (1.48, 1.79), respectively. Among the parental behavior factors, the mother’s exercise frequency had a strong association with the children’s MVPA and SLP, with OR values of 1.65 (1.50, 1.83) and 1.24 (1.13, 1.37), respectively; the mother’s screen time was inversely associated with the children’s ST with an OR value of 0.47 (0.44, 0.51). Conclusions: Different types of family environments were associated with the different levels of MVPA, ST and SLP among Chinese preschool children. In addition to the influence of parents’ education and family income, parents could also improve their children’s behaviors by providing a supportive family environment. The more of these factors presented in a family, the more likely it was for children to meet the guidelines. Therefore, for those families whose children’s health-related behaviors needed to be improved, the parents should create supportive family environments, such as by playing less on mobile phone and spending more time with children.

## 1. Introduction

Early childhood is a period of rapid development of the body’s physical and cognitive functions, and children also form habits and change lifestyles during this period of adaptation [1]. Developing health-related behaviors in this critical period could continue to effectively influence the course of their entire life [2]. Previous studies have explored the impact of health-related behaviors, such as physical activity (PA), sedentary behavior (SB), and sleep behavior (SLP), on various health-related outcomes among preschool children [3,4,5,6,7], which found that the moderate to vigorous physical activity (MVPA) could improve the ability of body movement and cardiovascular metabolism [3,4], while sedentary behavior led by long screen time behavior (ST) and insufficient sleep could harm preschool children’s physical and mental health [5,6].

Most of the current studies on 24-hour health-related behaviors previously focused on their relationship with sociodemographic factors and parental behavior factors. For example, a study evaluated the health-related behaviors of children aged 5.5 years old in Singapore [2], and found that ethnic Chinese, younger mothers, and mothers’ lower TV and sleep time were associated with higher ST compliance rates among children; while males, ethnic Malays, and higher mothers’ PA levels were associated with higher infants’ MVPA compliance rate; and older mothers were associated with infants’ adherence to sleep guidance. There was a study that specifically surveyed South African children in urban and rural areas and found that urban children’s health-related behaviors were higher than their rural peers, and children in high-income families had better health-related behaviors than low-income families [8]. At the same time, compared with parents from high-income families, those from low-income families believed that the length of screen time would not affect the health of their children. Therefore, it could be found that sociodemographic factors and parental behavioral factors could have an important impact on the three studied health-related behaviors of preschool children.

Previous studies explored the impact of multiple risk factors on adults’ health. For example, previous studies in the United States had found that adult obesity was determined by multiple factors, so it was necessary to intervene in obesity from multiple perspectives [9,10]. Similarly, recent studies began to address the combined effects and related factors of health-related behaviors in children [11,12]. The health-related behaviors of preschool children were coexistent and interrelated. However, within the 24 h of a day, the more time children spend on certain health-related behaviors, the less time they spend on other behaviors. Current research has also found links among different health-related behaviors. For example, one study conducted in China found that a long screen time could reduce children’s sleep time, but active physical activity could improve children’s sleep quality [13]. A Finnish study also found that with enough physical education classes, the higher the family’s SES, the longer the child’s sedentary time [14]. Thus, we tried to determine whether different influencing factors had different effects on a variety of health-related behaviors, or the difference in the types and numbers of influencing factors required different health-related behaviors to achieve standards for preschool children. Targeted promotion of one or more health-related behaviors of preschool children and optimization of the related intervention measures could be of important significance for this study. However, in the past, little research has explored the different combinations of the three types of health-related behaviors involving physical activity, screen time, and sleep behavior for preschool children during this period of behavioral plasticity.

The social-ecology model was regularly applied to explore the factors affecting physical activity in children. The social ecology model emphasized the interaction between human and social elements in the environment. The social-ecology model divided the ecological levels of society into the individual level, the interpersonal level, the community level, and the societal level [15]. For children aged 3–6, the influence of individual factors, family factors, and community factors were the most obvious [15]. Based on this model, we incorporated relevant factors for research. At the same time, when we looked up the relevant questionnaires, such as the Pre-PAQ (preschool-aged Children’s Physical Activity Questionnaire) [16], the HOME questionnaire [17], and the AHEMD-SR (affordances in the home environment for motor development self-report) questionnaire [18], on children’s health-related behaviors, we found that the survey indicators of these questionnaires were not only sociodemographic indicators and parental behaviors, but also investigated children’s supportive family environment and community factors. However, these indicators were rarely mentioned in the current analysis of children’s health-related behaviors. At the same time, the current research only considered a single factor among the three types of behaviors separately, or simply grouped them with “one movement behavior reaching the standard and two health-related behaviors reaching the standard” [19,20,21].

In 2017, Canada released the Canadian 24-Hour Movement Guidelines for the Early Years, the world’s first integrated health-related behavior guidelines that recommend the amount and intensity of physical activity, sleep, and limits to sedentary time [22]. Similarly, World Health Organization (WHO) adopted the same standard in its guidelines for PA, SB, and SLP for children under 5 Years of Age released in 2019 [23]. However, according to recent studies from Finland, Canada, and Australia, only 12.7% to 24% of children met the movement guidelines in their early years [21,24,25]; therefore, promoting health-related behaviors in preschool children was still an important long-term task. In 2020, China also proposed the corresponding “Guidelines for Exercise for Preschool Children (3–6 Years)” [26], and its recommended standards were the same as those of the WHO. At present, there is a study in China that has investigated the current status of children’s health-related behaviors in some areas. For example, Chang’s survey of children’s health-related behaviors in Shanghai, China found that the MVPA compliance rate of preschool children in this area was 41.7% [27]. However, few studies have explored the status quo of China’s preschool children’s health-related behaviors in a nationwide survey.

Therefore, the purpose of this study was to investigate the compliance rate of three health-related behaviors among Chinese preschool children using a national representative survey and to explore how supportive family environment, parental behavior, sociodemographic and community factors influence children’s health-related behavior comprehensively based on the social-ecology model. The present study could help provide strong evidence and clear health promotion interventions implemented in early childhood, which could produce benefits for preschool children’s physical and mental health in the future.

## 2. Materials and Methods

### 2.1. Study Setting and Participants

The current study was extracted from a large nationally representative survey, “Preschool Children National Fitness Status Survey”, by the General Administration of Sport of China. The purpose of this survey was to examine three health-related behaviors among Chinese preschool children. The samples were chosen from 31 provinces by use of a stratified random sampling procedure involving three stages. For the randomization of the sampling, the survey team had cooperation with a technology company to develop random sampling software. At the same time, corresponding sampling replacement rules had been formulated. The scheme for sample replacement was based on preschool children of the same sex and age in the same area, ensuring that the sample reflected the regional characteristics and the overall level of preschool children’s behaviors. The inclusion criteria of the sample were individuals who had lived in the designated local study areas for at least six months. We conducted interviews with their primary caregivers to collect the children’s health-related behaviors and other relevant information. However, if their primary caregivers had cognitive, language, or other impairments and were unable to complete our interview, it would lead us to collect inaccurate information and affect the investigation results. Therefore, the exclusion criteria were the preschool children whose primary caregivers had cognitive and language impairments.

**Figure 1:**
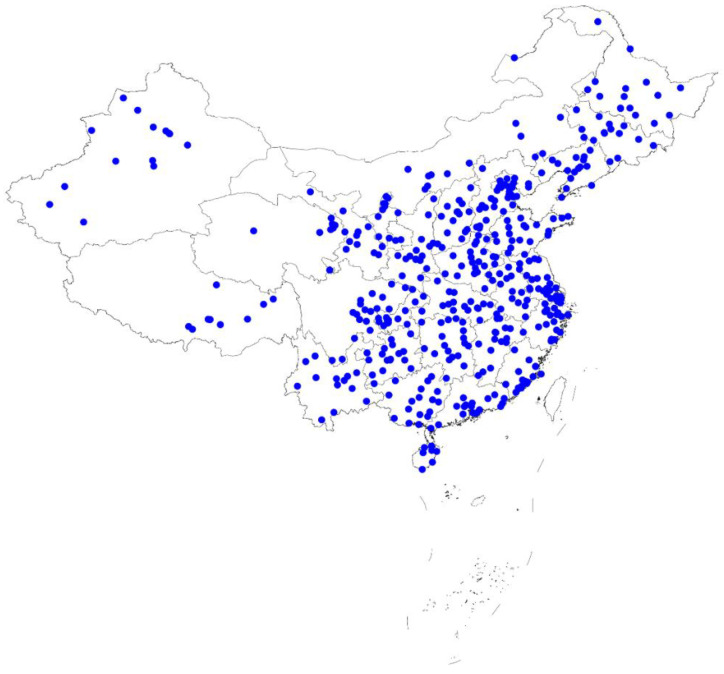
The distribution of the sample counties in China.

Preschool children aged 3–6 years old from 31 provinces were covered in 471 counties (districts) and 5760 villages (residential) committees in China. A total of 10–20 counties (districts) were randomly selected in each province, 13 villages (neighborhood) committees were randomly selected in each county, and two children aged 3–6 years were selected in each village (neighborhood) committee. The sample size was calculated using the formula *N* = *deff* × *T*^2^ × *p*(1 − *p*)/*d*^2^. For people aged 3–6, the *deff* value is 2, the *T* value is 1.96, the relative error is 15%, and the *p* value is estimated as 0.5 [28]. It was estimated that 11,520 children would be investigated. The distribution map of the sample counties (districts) is shown in Figure 1. Each point represented a county (district) selected in this survey. The survey covered all provinces in mainland China with a very wide range of distribution. Children in China were well represented throughout the country. The survey started in September 2020 and was completed in November 2020. The local government downgraded the response level of the COVID-19 to level III in May 2020 [29]. Therefore, when the data was collected in this study, the COVID-19 had little impact on the daily lives of Chinese people. At the same time, we had also carried out corresponding sample replacements for a small number of families who were not willing to be surveyed. Finally, the effective sample size included in the statistical analysis was 10,967 preschool children. The survey churn rate was 5.3%, which included families who were unwilling to be surveyed due to COVID-19 and other reasons. The survey was conducted after the respondent agreed to be surveyed and signed a written informed consent. The legal representatives of the children and the children themselves provided written informed consent. Full ethical approval was obtained from the China Institute of Sport Science, Beijing, China (CISS-2019-10-29).

### 2.2. Procedures

The survey was carried out by the national research team to make relevant project plans. Provincial and municipal sports bureaus and scientific researched institutes conduct investigator training. The investigators at the monitoring points of all districts and counties conducted questionnaire surveys. The information of the interviewees at the monitoring points came from the local statistical bureau. After each monitoring point summarized the information and entered it into the data information collection platform of the project team, randomly selected samples that met the requirements, and conducted a face-to-face survey after obtaining permission through telephone contact. The interviewers of the questionnaire were trained by the sports bureaus of each province to participate in the survey. Face-to-face interviews were conducted in neighborhoods throughout the designated study locations. The interviewers who had received training before would ask questions and input the data into the computing platform. The questionnaire used in this study was compiled by the China Institute of Sports Science [28]. Each interview lasted approximately one hour per participant. The responses to the questionnaires were made by the participants’ parents, lasting approximately one hour per participant in each interview. At the same time, quality control was carried out through telephone return visits and on-site recording during the investigation.

### 2.3. Measures

After the investigator was trained and familiar with the questionnaire, they brought the relevant documents and entered the household with the permission of the interviewee to conduct a face-to-face survey. At the same time, the investigation team leader was responsible for the process management and quality control of the monitoring point, including organization, sample replacement, and response to emergencies. Quality control was mainly carried out through recording quality control, which was divided into district and county level, provincial level and central level control. The unqualified results will be fed back to the monitoring point for quality improvement. All indicators were obtained by asking parents of preschool children through questionnaires, including the child’s height and weight.

The questionnaire was self-designed concerning the Pre-PAQ [16], the HOME questionnaire [17], the AHEMD-SR questionnaire [18], and the previous national survey questions [30,31]. The China Institute of Sport Science launched two rounds of expert opinion consultation. The first round of the expert survey passed the five-point Likert scale to evaluate the importance of the indicators, and the preliminary screening of the indicator system was completed. In the second round of the survey, the adjusted results and questions of the first round of the survey were fed back to the experts, who were asked to continue to evaluate the importance of the revised index evaluation system to determine the final evaluation index system. The Cronbach’s alpha coefficient of the total questionnaire was 0.82. The questionnaire consisted of five parts, including the average daily duration of three health-related behaviors, sociodemographic factors, supportive family environmental indicators, parental behavior indicators, and community environment indicators. The sociodemographic indicators mainly included the child’s gender, age, urban and rural areas, left-behind, parents’ education level, and total annual household income; the supportive family environmental indicators mainly included parents’ attitudes towards sports activities, the number of sports equipment at home, and parents’ time with their children on weekends and children’s athletic ability evaluated by parents; parent’s behavioral factors mainly included parents’ obesity status, parent’s exercise frequency, and parents’ time spent on electronic screens on weekends; community environment indicators mainly included parents’ evaluation of whether the surrounding environment met sports activities and the number of surrounding factors that hindered going out, etc. The measurement methods of MVPA, ST, and SLP are shown below, and the specific items and scoring methods of other indicators are shown in Attached Table A1.

#### 2.3.1. Moderate to Vigorous Physical Activity

MVPA was determined by asking parents to recall the child’s daily life in the past week, investigating the activities of moderate-intensity and above in the morning, afternoon, and evening. Each period was divided into five levels: “no exercise at all”, “1–15 min”, “16–30 min”, “31–60 min”, and “more than 60 min”. By informing parents that the child sweats slightly and breathes faster, MVPA was used to control the intensity of the activity under investigation.

#### 2.3.2. Screen Time Behavior

ST was determined by asking parents to recall the average daily screen time of their children in the past one week. The ST was divided into seven levels: “not looking at the electronic screen”, ”1–30 min”, “31–60 min”, “61–90 min”, ”91–120 min”, “121–180 min”, and “181 min and above”.

#### 2.3.3. Sleep Behavior

SLP was determined by asking parents to recall the average daily sleep time of their children in the past one week. The SLP was divided into 7 levels: “less than 8 h”, “8–9 h”, ”9–10 h”, “10–11 h “, “11–12 h”, ”12–13 h”, “more than 13 h”.

#### 2.3.4. Three Health-Related Behaviors Standards

The evaluation method of compliance rate: the WHO recommended that preschool children take at least 60 min of MVPA per day, no more than 1 h of ST per day, and at least 10 h of SLP per day. According to the standard, children who met the recommendations were regarded as a population with positive and healthy behaviors, and those who did not meet the recommendations were regarded as a population with negative and unhealthy behaviors [23]. China’s recommended standards were the same as those of the WHO [26]. A pragmatic approach was used with regards to the duration component, as parents tend to over-report MVPA and under-report ST [32]. We took the middle value of SLP.

**Figure 2:**
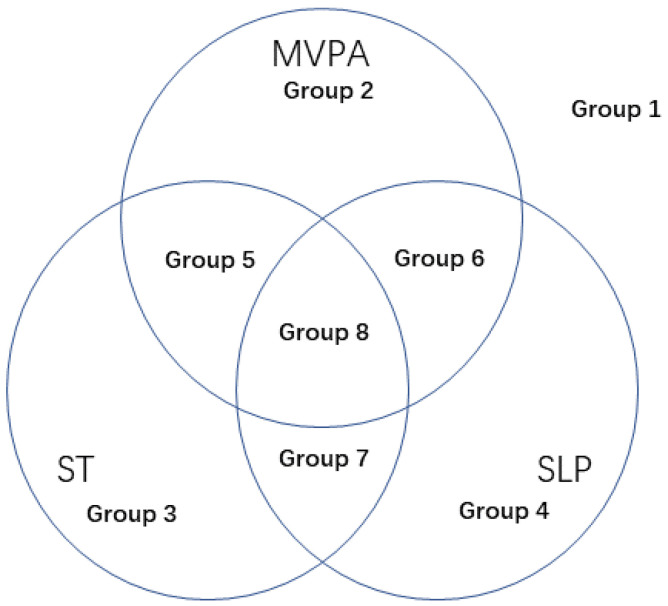
The eight groups for three health-related behaviors. Notes: Group 1 represents preschool children who did not meet the three health-related behaviors; Group 2 represents preschool children who only met the MVPA guideline; Group 3 represents preschool children who only met the ST guideline; Group 4 represents preschool children who only met the SLP guideline; Group 5 represents preschool children who met the MVPA and ST guidelines; Group 6 represents preschool children who met the MVPA and SLP guidelines; Group 7 represents that preschool children who met the ST and SLP guidelines; and Group 8 represents preschool children who met all three health-related behaviors’ guidelines.

As shown in Figure 2, the combination of three health-related behaviors was divided into eight groups: based on the compliance rate of MVPA, ST, and SLP, a total of eight health-related behavior groups were obtained: preschool children who did not meet the three health-related behaviors (group 1), only MVPA met the guideline (group 2), only ST met the guideline (group 3), only SLP met the guideline (group 4), MVPA and ST met the guidelines (group 5), MVPA and SLP met the guidelines (group 6), ST and SLP met the guidelines (group 7), and all three health-related behaviors met the guidelines (group 8).

### 2.4. Statistical Analysis

The descriptive statistics (mean and standard deviations) were used to describe numerical variables (e.g., age, height, weight, etc.). Descriptive statistics of frequency and rate data were used for categorical variables (e.g., gender, urban and rural dwelling, income level, educational level, occupation, etc.). We used logistic regression to analyze the factors affecting preschool children’s MVPA, ST, and SLP. The dependent variable was whether or not the three guidelines were met. The independent variables included social demographic factors, family environmental indicators, parental behavior indicators, and community environmental indicators. We used logistic regression to analyze the influencing factors of the eight combinations of health-related behaviors shown in Figure 2. Group 1 was used as the control group and compared with the other seven groups as dependent variables. The independent variables included social demographic factors, family environmental indicators, parental behavior indicators, and community environmental indicators. At the same time, we considered controlled and uncontrolled influencing factors related to the child themselves, including the child’s age, gender, height, and weight. Aside from influencing factors analysis, we considered whether the three health-related behaviors met guidelines. This was a two-tailed test with the significance level set to 0.05. The data were analyzed with SPSS software 22.0 (IBM Inst., Chicago, IL, USA).

## 3. Results

### 3.1. The Basic Characteristics of the Survey Sample

The demographic and descriptive characteristics and statistics of family environment, parental behavior, and community environment indicators of the participants are shown in Table 1. The sample was comprised of 5947 (54.2%) boys and 5020 (45.8%) girls with the mean age of 4.82(±1.05). The mean height was 110.77(±10.82) cm, and the mean weight was 21.09(±5.20) kg. Among them, 60.6% of the participants came from urban areas and 39.4% of them were rural peers. About 30.7% of the participants’ fathers and 30.1% of the participants’ mothers had a college of professional training degree or above, and 70.1% of the participants’ families had a total annual household income of less than 80,000 yuan.

### 3.2. The Current Status of the Three Health-Related Behaviors

Table 2 shows how the three health-related behaviors of Chinese preschool children met the requirements of the guidelines. The proportion of Chinese preschool children who met the MVPA, ST, and SLP guidelines were 62.3%, 52.8%, and 53.8%, respectively. Among them, boys’ three behaviors compliance rates were 62.1%, 52.1%, and 53.6%, and they were 62.5%, 53.6%, and 53.9% in girls. Girls’ behavior compliance rates were higher than boys. There was no significant difference in the compliance rates of various behaviors between boys and girls. The compliance rates of the three behaviors of urban preschool children were 63.1%, 53.3%, and 54.4%, and they were 58.6%, 52.0%, and 52.7% in rural children. The compliance rates of all behaviors of urban preschool children were higher than those of rural preschool children. There was only a significant difference in MVPA, with a higher compliance rate in urban than rural children, and there was no significant difference in the other two behaviors’ compliance rates between sexes and urban/rural areas.

**Figure 3:**
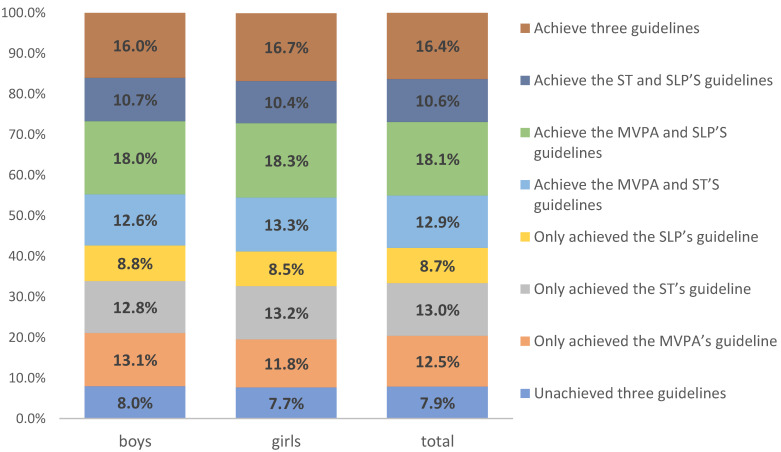
The proportions of the eight groups in the respondents.

According to whether the three behaviors met the requirements of the guidelines, they could be divided into eight groups. Figure 3 shows the proportions of the eight groups in the respondents. Through comparison, the proportions of the eight groups were relatively close, regardless of whether it was the whole group or boys and girls. The behavior combination that accounts for the largest proportion was the MVPA and SLP achieved guidelines combination (total: 18.1%, boys: 18.0%, and girls: 18.3%), and the second most common behavior group was the combination of MVPA, ST, and SLP achieved guidelines (total: 16.4%, boys: 16.0%, girls: 16.7%). The behavior combined with the lowest proportion was the combination in which none of the three behaviors met guidelines. (total: 7.9%, boys: 8.0%, girls: 16.4%).

### 3.3. Related Influencing Factors of Three Health-Related Behaviors

Table 3 shows the independent influencing factors of three health-related behaviors of children through the logistics regression model. Among the sociodemographic factors, total annual household income had the greatest impact on children’s MVPA and ST, with OR values of 1.59 (1.44, 1.75) and 0.75 (0.68, 0.83), respectively; age indicators had the most significant impact on children’s SLP with an OR value of 0.79 (0.76, 0.82). Among the supportive family environmental factors, parents’ time with their children on weekends had the most significant impact on children’s MVPA, ST, and SLP. The OR values were 2.18 (1.97, 2.40), 0.69 (0.63, 0.76), and 1.62 (1.48, 1.79). Among the parental behavior factors, the mother’s exercise frequency had the greatest impact on the children’s MVPA and SLP, with OR values of 1.65 (1.50, 1.83) and 1.24 (1.13, 1.37) respectively; the mother’s time spent on electronic screens on weekends had a significant impact on the children’s ST, and its OR value was 0.47 (0.44, 0.51). Among the community environment indicators, whether the surrounding environment meets sports activities had a significant impact on children’s MVPA and ST, and the OR values were 1.42 (1.31, 1.53) and 1.23 (1.14, 1.33), respectively; and whether there were obstacles in the surrounding environment had the most significant impact on young children’s SLP, with an OR value of 1.14 (1.05, 1.22).

In Table 4, the “all non-meet-guideline group” among the eight combinations of three health-related behaviors was used as the control group, and the remaining seven groups were subjected to binary logistics regression analysis, and the OR values obtained were filled in the table for drawing. The regression analysis chart of controlling the children’s demographic factors is shown in Attached Table A2 and Table A3. Comparing the three groups of preschool children with the groups where only one indicator met the guideline and the “all non-meet-guideline group”, compared in Table 3, the number of influencing factors decreased to 8 (MVPA), 12 (ST), and 5 (SLP).

The influencing factors of MVPA were mainly family environmental factors, parental behavioral factors, and community environmental factors, and the influential factors of ST were mainly social demographic factors and parental behavioral factors. The number of factors affecting SLP was relatively few, and the distribution of types was relatively even. Comparing the three groups of preschool children who met the two health-related behavior guidelines with the “all-meet-guideline group “, it was found that father’s education level, mother’s education level, and total annual household income of the MVPA and ST meeting guidelines group were the main influencing factors. The main influencing factors were the father’s education level, mother’s education level, father’s obesity status, mother’s obesity status, the father’s time spent on electronic screens on weekends, and the mother’s time spent on electronic screens on weekends in the MVPA and SLP meeting guidelines group. Comparing the ST and SLP meeting guidelines group with the “all-meet-guideline group”, it was found that father’s education level, mother’s education level, the number of sports equipment, and the parents’ time with their children on weekends were the main influencing factors.

In Table 4, when comparing the three categories of preschool children with only one kind of health-related behavior that met the guideline with the “non-meet-guideline group”, children’s athletic ability evaluated by parents (OR = 1.34), and the number of sports equipment (OR = 1.60), the father’s exercise frequency (OR = 1.38) had a significant impact on only the MVPA meeting guideline group. The father being obese (OR = 0.75), the father’s time spent on electronic screens on weekends (OR = 0.53), and the mother’s time spent on electronic screens on weekends (OR = 0.46) had a significant negative impact on only the ST meeting the guideline group. When comparing the three categories of preschool children whose two kinds of health-related behaviors met the guideline with the “non-meet-guideline group”, it was found that the above-mentioned influencing factors had an impact on preschool children with other health-related behaviors (for example, only the MVPA meeting guideline group with the MVPA and ST meeting the guidelines group or the MVPA and SLP meeting guidelines group) had a significant impact.

## 4. Discussion

This study was the first to cover all provinces in Mainland China with a large sample of 3–6-years-old children collecting health-related behaviors and related influence factors. The study revealed the current compliance rate with WHO standards in Chinese preschool-aged children. We also found that the different effects on preschool-aged children’s three health-related behaviors from sociodemographic factors, supportive family environment, parental health-related behaviors, and community environment. This study further explored the significant influencing factors of the three health-related behaviors of preschool children, and provided references for parents or related experts to promote one or more health-related behaviors in children and optimize intervention methods according to specific conditions.

### 4.1. The Current Status of Chinese Preschool Children’s Health-Related Behavior Compliance Rate and Related Sociodemographic Factors

Due to the lack of similar cross-sectional studies in this field in China, we referred to recent international studies with similar sampling and adopted the same standard in this study. According to a cross-sectional survey of 778 children in Finland conducted by Leppänen in 2019, 85% of Finnish children met the MVPA, 35% met the ST, 76% met the SLP, and 24% of the total sample met all three criteria [18]. In another study conducted by Cliff in 2017, of 248 Australian children that participated in the survey, 93.1% met MVPA, 17.3% met ST, 88.7% met SLP, and 14.9% met all three criteria [25]. In 2017, Chaput surveyed 803 Canadian children with a compliance rate of 61.8% for MVPA, 24.4% for ST, 83.9% for SLP, and 12.7% for all above three behaviors [24]. Comparing with the studies mentioned above, the compliance rate of MVPA and SLP in Chinese children was slightly lower, while the compliance rate of ST was significantly higher than that in some other developed countries’ studies. This suggested that more attention needed to be paid to the deficiencies of preschool children’s MVPA and SLP with a formulation of corresponding plans to improve children’s health-related behaviors.

We found urban children had better health-related behavior performance than rural children. Compared with rural children, urban children had better objective conditions to meet the movement behavior guidelines. This finding was consistent with a South African study on the health-related behaviors of urban and rural children [19]. According to that study, urban children under strict requirements by their parents were more likely to comply with health-related behaviors than rural children. This was related to the status of health education in rural China. A Chinese study found that although the health level of children in rural China and the health education level of parents have improved in recent years [33], there were still structural differences. There were a considerable number of left-behind children in rural China. They were usually raised by grandparents. The lack of health education for left-behind children in rural China caused by this phenomenon was even more obvious, which in turn affects the health behaviors of rural children [34]. At the same time, left-behind children in China had more access to electronic screens than in the past, but were not restricted by their parents. Therefore, they were prone to developing unhealthy lifestyles, especially screen time behaviors. This phenomenon had also been similarly discovered in a Mexican study [35].

We also found that the family income’s correlation with MVPA was opposite to that with ST. The higher the family income, the easier the children’s MVPA to meet the standard, while the ST was more difficult to meet the standard. This was because high-income families could provide children with adequate equipment—sports equipment or electronic screen equipment—which would shape children’s behavior in an objective environment [36]. This phenomenon might be more obvious among adolescents [37]. Other current studies have also found that the better the family’s financial status. More affluent parents were more likely to transport their children to other activity opportunities than parents from the lower-income brackets [38]. The survey found that parental education was a key factor that affects the transition from two behaviors to three behaviors in this group. Families with highly educated parents were more willing to keep their children in a state of comprehensive health. Parents’ education level would affect parents’ support for children’s participation in physical activities and indirectly affect children’s participation in physical activity [39]. It was suggested that children of parents with low educational backgrounds should be the focus of the intervention to help children shape healthy activity behaviors and lifestyles. Therefore, the urban and rural level, family income, and parents’ educational background affected the composition of the family environment and had an important impact on the early development of children. However, the above indicators were indicators that the family would not change in the short term, and it was difficult for parents to improve these indicators to promote the development of children’s healthy behaviors [40,41].

### 4.2. Related Influencing Factors of Three Health-Related Behaviors in Different Groups

However, for preschool children, the relevant sociodemographic factors were difficult to change in a short period. As such, we would need to pay more attention to some influencing factors that parents could easily change in the family environment, such as the supportive family environmental factors and parents’ behavioral factors.

The MVPA of preschool children was greatly affected by the environmental factors of supportive family sports, the frequency of parental exercises, and the environmental factors of the community. The parents of only MVPA behaviors meeting the guideline group paid more attention to their children’s athletic ability and peacetime exercise, and actively provided their children with a good exercise environment [42], but they might not pay enough attention to other influencing factors. The other health-related behaviors were not up to standard. For example, in the ST+ SLP compliance group, the importance of parents’ attention to sports and the objectively provided items for children’s sports were the keys to children’s promotion of MVPA behavior when the other two behaviors meet the standards. On the contrary, the parents had other conditions. Under the circumstances, if children were not provided with enough sports equipment, or the time spent with children during exercise was too little, it would affect the achievement of children’s MVPA behavior [43]. When the number of health-related behaviors that met the criteria was the same, the environmental factors of supportive family sports had a greater impact on people with MVPA that met the criteria. It could be considered that we could improve the MVPA of preschool children by focusing on improving the environmental factors of supportive family sports.

The ST of young children was greatly affected by sociodemographic factors, the degree of parental obesity, and parental screen time. This was similar to the results of some studies [2,44], when the daily behavior of young children’s parents had health risks such as obesity, sitting quietly, and less movement. When there was a problem, it would affect the child’s SB and ST, making it difficult for the child’s ST to reach health standards. Among the parental behavior factors, the higher the mother’s exercise frequency, the easier it was for the child’s MVPA and SLP to meet the standards, and the longer the mother’s screen time, the more difficult it was for the child’s ST to meet the standards. Studies also found that mothers’ higher MVPA and lower ST were associated with children’s higher MVPA and ST compliance rates [45]; other cross-sectional studies have reported direct correlations between mothers and children in behavior and sleep patterns [2]. Therefore, by improving the parent’s MVPA or ST, the child’s health-related behaviors could be correspondingly improved. Although this study lacked the data to investigate mothers’ sleep time, it did not rule out the important influence of mother’s sleep on children’s sleep.

Community environment factors also had a significant influence on children’s health-related behaviors. This study specifically surveyed parents for community surrounding environment such as venue space, green conditions, safety situation, and peer accompany. We also surveyed the current limitations of the community environment, such as lack of equipment, high population density, etc. Several studies have proved that safety [15], population density [46], and venue space [15] are important factors regarding health-related behaviors, especially MVPA. Improving the community environment to assure parents can further improve children’s health-related behaviors.

### 4.3. The Status of the Combined Effects of Three Health-Related Behaviors

This survey found that as the number of children’s health-related behaviors increased, the types and numbers of related influencing factors also increased. It reminded us that we can improve children’s certain health-related behaviors by improving a few influencing factors of children. However, when our goal is to reach the standards for more children’s health-related behaviors, there are more influencing factors that need to be improved. The influencing factors for specific health-related behaviors have been mentioned above. Our results were akin to the results of a Finnish study investigating the relationship between health-related behaviors and obesity in preschool children [21]. They found that meeting two or three health-related behaviors guidelines instead of none or one was associated with lower body mass index and waist circumference. These findings support the theory that these three health-related behaviors were codependent, and thus, there was a great need for interventions that attempt to promote compliance of more than one health-related behavior at a time. Although the purpose and influencing factors of the research were slightly different from our survey, the direction of the conclusion was the same.

In addition, this survey also found some influencing factors such that parents’ evaluation of their children’s exercise capacity, the number of sports equipment at home, and the father’s exercise frequency had a significant impact on the health-related behaviors of those who only meet the MVPA standard. However, when we compared the two movement behavior standards with those who did not meet the standards, we find that the above-mentioned influencing factors had an impact on the MVPA behavior and significantly on the preschool children who met the other movement behavior standards (MVPA and ST compliance group or MVPA and SLP compliance group). The same phenomenon also occurred in preschool children whose only ST compliance group and ST and other health-related behaviors met the standards (MVPA and ST compliance group or ST and SLP compliance group), whether the father was obese, the father’s screen time, and the mother’s screen time. We found that the influencing factors with this characteristic were close to the main influencing factors of the individual health-related behavior mentioned above. Children were in the behavioral shaping period of human growth and development. For example, other studies have shown that training and improving children’s displacement skills in early childhood could also enhance children’s physical control skills [47], and for children’s fitness and other indicators, there were similar findings [48,49]. Therefore, based on the survey results of this study, it could be inferred that through the improvement of the above factors raise a health-related behavior could promote other health-related behaviors while improving. The influence of influencing factors on movement behavior was a survey study with large sample size and very wide coverage. It not only helped families and interveners to effectively promote children’s health activities, but also helped the government to formulate policies related to children’s health, assess the status of children’s health activities, and provide important information for the promotion of children’s healthy lives in China and even internationally.

### 4.4. Strengths and Limitations

The strengths of the present study were that this study was the first large-scale population health activity behavior survey of Chinese children. A face-to-face questionnaire survey was used to investigate the health-related behaviors of more than 10,000 children and their parents in 31 provinces in China, and extensive assessments including sociodemographic factors, supportive family environment factors, parental behavior factors, community environmental factors, etc.

Similarly, we also admit that this study had certain limitations. First, this study was a cross-sectional study. The national survey on which this study was based is a survey conducted by different investigators in different communities and households at the same time point, in different regions, and may have measurement bias. However, the investigators have been systematically trained by relevant experts from provincial and municipal sports bureaus and scientific research institutes. Only trained investigators can participate in our investigation. Therefore, we have minimized this measurement bias through strict quality control of investigators. Secondly, the surveyed indicators (such as MVPA, ST, SLP, etc.) were obtained through questionnaire surveys instead of instrumental measurements. The self-reported MVPA value is usually higher, and the ST value is usually lower [32]. This caused the results obtained to be carefully considered when compared with other studies, but this study had made up for this shortcoming as much as possible through large samples. At the same time, the survey of this research will be affected by social expectations, and respondents may give feedback with high social expectations. This may overestimate our results. However, this research mainly explores the number and intensity of factors that affect health-related behaviors. Even if there is an overestimation, the results of this study will help us identify the beneficial influence factors for children’s health-related behaviors. COVID-19 had a small impact on our investigation process. However, through China’s control of COVID-19 and the standardized sample replacement of families who were unwilling to be surveyed in this study, the impact of COVID-19 on the results of this study was limited. In addition, the recall bias caused by the primary caregiver’s response to the questionnaire could be unavoidable, but we used face-to-face questioning method, which significantly reduced the effect of recall bias on our results. Finally, due to the self-constructed questionnaire according to the realistic implementation, the lack of enough reliability and validity data was an important weakness of this study and needs to be further evaluated. Finally, the subjects of this study only include Chinese residents. The conclusion may have certain limitations on the extrapolation of children with different educational backgrounds, cultural backgrounds, and behavior habits in other countries. However, this study provides information that enables international comparison of children’s compliance with health-related behavior guidelines and related influencing factors, and can provide important information for other countries and regions in the world to promote children’s healthy lives.

## 5. Conclusions

This study found that it was necessary to increase the compliance rate of MVPA, ST, and SLP among Chinese preschool children. Different types of family environments were associated with the different levels of their children’s MVPA, ST, and SLP. In addition to the influence of parents’ education and family income, parents can also improve their children’s behaviors by providing a supportive family environment. We found that each type of children’s health behavior was associated with different family factors. The more of these factors presented in a family, the more likely it was for children to meet the guidelines. Therefore, for those families whose children’s health-related behaviors needed to be improved, the parents should act as soon as possible, by, for example, playing less with mobile phones and spending more time with their children in their leisure time, to improve the health of their children.

## Figures and Tables

**Table 1:** Demographic information of the participants; mean (SD) or number (percent).

Characteristics	Boys (N = 5947)	Girls (N = 5020)	Total (N = 10,967)
Age	4.82 (1.06)	4.81 (1.04)	4.82 (1.05)
Height	111 (10.96)	110.5 (10.64)	110.77 (10.82)
Weight	21.39 (5.24)	20.74 (5.13)	21.09 (5.20)
Urban and rural			
Urban	3628 (61.0)	3018 (60.1)	6646 (60.6)
Rural	2319 (39.0)	2002 (39.9)	4321 (39.4)
Left-behind			
No	5320 (89.4)	4514 (89.9)	9834 (89.7)
Yes	628 (10.6)	506 (10.1)	1133 (10.3)
Father’s Educational level			
I	1819 (30.6)	1547 (30.8)	3366 (30.7)
II	1639 (27.6)	1388 (27.6)	3027 (27.6)
III	2489 (41.9)	2085 (41.5)	4574 (41.7)
Mother’s Educational level			
I	1793 (30.1)	1511 (30.1)	3304 (30.1)
II	1524 (25.6)	1301 (25.9)	2825 (25.8)
III	2630 (44.2)	2208 (44.0)	4838 (44.1)
Income (total annual household income)			
Over 80,000 yuan	1792 (30.1)	1491 (29.7)	3283 (29.9)
40,000–80,000 yuan	2226 (37.4)	1807 (36.0)	4033 (36.8)
Less than 40,000 yuan	1929 (32.4)	1722 (34.3)	3651 (33.3)
Children’s athletic ability evaluated by parents			
Pretty good	3047 (51.2)	2478 (49.4)	5525 (50.3)
General	2900 48.8)	2542 (50.6)	5442 (49.7)
Sports equipment			
2 or more Sports equipment	3115 (52.4)	2376 (47.3)	5491 (50.0)
Less than 1 Sports equipment	2832 (47.6)	2644 (52.7)	5476 (50.0)
Parents’ time with their children on weekends			
More than 4 h a day	1715 (28.8)	1487 (29.6)	3202 (29.2)
2 to 4 h a day	2281 (38.4)	1787 (35.6)	4068 (37.1)
Less than 2 h a day	1951 (32.8)	1746 (34.8)	3697 (33.7)
Parents’ attitudes towards sports activities			
Positive	1960 (33.0)	1551 (30.9)	3511 (32.0)
Neutral	1746 (29.4)	1480 (29.5)	3226 (29.4)
Negative	2241 (37.7)	1989 (39.6)	4230 (38.6)
Father’s obesity status			
Obesity	1384 (23.3)	1212 (24.1)	2596 (23.7)
Normal	4563 (76.7)	3808 (75.9)	8371 (76.3)
Mother’s obesity status			
Obesity	808 (13.6)	731 (14.6)	1539 (14.0)
Normal	5139 (86.4)	4289 (85.4)	9428 (86.0)
Father’s exercise frequency			
3 or more times a week	1298 (21.8)	1078 (21.5)	2376 (21.7)
1 to 3 times a week	1968 (33.1)	1572 (31.3)	3540 (32.3)
Less than 1 a week	2681 (45.1)	2370 (47.2)	5051 (46.1)
Mother’s exercise frequency			
3 or more times a week	1386 (23.3)	1208 (24.1)	2594 (23.7)
1 to 3 times a week	1857 (31.2)	1536 (30.6)	3393 (30.9)
Less than 1 a week	2704 (45.5)	2276 (45.3)	4980 (45.4)
Father’s time spent on electronic screens on weekends			
2 or more hours per day	2723 (45.8)	2344 (46.7)	5067 (46.2)
Less than 2 h a day	3224 (54.2)	2676 (53.3)	5900 (53.8)
Mother’s time spent on electronic screens on weekends			
2 or more hours per day	2412 (40.6)	2032 (40.5)	4444 (40.5)
Less than 2 h a day	3535 (59.4)	2988 (59.5)	6523 (59.5)
Whether the surrounding environment meets sports activities			
Enough	3312 (55.7)	2755 (54.9)	6067 (55.3)
Something is lacking	2635 (44.3)	2265 (45.1)	4900 (44.7)
Surrounding factors that hinder going out			
Almost none	3261 (54.8)	2690 (53.6)	5951 (54.3)
A few	2686 (45.2)	2330 (46.4)	5016 (45.7)

Notes: I: College for professional training, bachelor’s degree or above; II: High school, technical secondary school or technical school; III: Junior middle school or below.

**Table 2:** The rates of three health-related behaviors of Chinese preschool children that met the requirements of the guidelines.

	MVPA	ST	SLP
Total	62.3% (61.4%,63.2%)	52.8% (51.8%,53.8%)	53.8% (52.8%,54.7%)
Boys	62.1% (60.9%,64.5%)	52.1% (50.8%,53.4%)	53.6% (52.3%,54.8%)
Girls	62.5% (60.2%,63.9%)	53.6% (52.8%,55.0%)	53.9% (52.6%,55.3%)
Urban	63.1% (61.9%,64.3%)	53.3% (52.1%,54.5%)	54.4% (53.2%,55.6%)
Rural	58.6% * (57.1%,60.0%)	52.0% (50.5%,53.5%)	52.7% (51.2%,54.2%)

Notes: * *p* < 0.05 vs. urban preschool children.

**Table 3:** The independent influencing factors of three health-related behaviors of children.

		MVPA	ST	SLP
		OR (95%CI)	OR (95%CI)	OR (95%CI)
Sex (ref: girls)				
Boys		0.98 (0.91,1.06)	0.94 (0.87,1.01)	0.99 (0.92,1.06)
Age		0.96 (0.93,1.00) *	0.96 (0.93,1.00) *	0.79 (0.76,0.82) **
Urban and rural (ref: rural)				
Urban		1.21 (1.12,1.31) **	1.05 (0.98,1.14)	1.07 (1.00,1.16)
Height		1.00 (1.00,1.01)	1.00 (0.99,1.00)	0.98 (0.98,0.99) **
Weight		0.99 (0.98,1.00) *	0.99 (0.99,1.00)	0.96 (0.95,0.97) **
Left-behind (ref: Yes)				
No		1.13 (1.00,1.28)	1.31 (1.16,1.49) **	0.89 (0.78,1.00)
Father’s Educational level (ref: III)				
I		1.48 (1.35,1.63) **	1.08 (0.99,1.18)	1.18 (1.08,1.29) *
II		1.13 (1.03,1.24) *	0.98 (0.90,1.08)	0.97 (0.89,1.06) *
Mother’s Educational level (ref: III)				
I		1.41 (1.29,1.55) **	1.09 (1.00,1.19)	1.14 (1.04,1.25) **
II		1.09 (0.99,1.19)	0.99 (0.90,1.09)	0.94 (0.86,1.04)
Income (total annual household income) (ref: Less than 40,000 yuan)				
Over 80,000 yuan		1.59 (1.44,1.75) **	0.75 (0.68,0.83) **	1.35 (1.23,1.49) **
40,000–80,000 yuan		1.36 (1.25,1.49) **	0.79 (0.72,0.87) **	1.09 (0.99,1.19)
Children’s athletic ability evaluated by parents (ref: general)				
Pretty good		1.44 (1.34,1.56) **	1.22 (1.13,1.32) **	1.11 (1.03,1.20) **
Sports equipment (ref: Less than 1 Sports equipment)				
2 or more Sports equipment		1.83 (1.68,1.98) **	1.08 (1.00,1.16)	1.21 (1.12,1.31) **
Parents’ time with their children on weekends (ref: Less than 2 h a day)				
More than 4 h a day		2.18 (1.97,2.40) **	0.69 (0.63,0.76) **	1.62 (1.48,1.79) **
2 to 4 h a day		1.85 (1.68,2.02) **	0.63 (0.57,0.70) **	1.36 (1.25,1.49) **
Parents’ attitudes towards sports activities (ref: Negative)				
Positive		1.94 (1.76,2.22) **	1.39 (1.24,1.49) **	1.24 (1.13,1.36) **
Neutral		1.56 (1.45,1.75) **	1.19 (1.08,1.30) **	1.16 (1.06,1.27) **
Father’s obesity status (ref: Normal)				
Obesity		1.01 (0.92,1.10)	0.76 (0.70,0.83) **	1.01 (0.93,1.10)
Mother’s obesity status (ref: Normal)				
Obesity		0.96 (0.86,1.07)	0.75 (0.67,0.84) **	0.99 (0.88,1.10)
Father’s exercise frequency (ref: Less than 1 a week)				
3 or more times a week		1.53 (1.39,1.70) **	1.41 (1.278,1.56) **	1.19 (1.08,1.31) *
1 to 3 times a week		1.26 (1.15,1.38) **	1.26 (1.16,1.38) **	0.91 (0.84,0.99)
Mother’s exercise frequency (ref: Less than 1 a week)				
3 or more times a week		1.65 (1.50,1.83) **	1.30 (1.18,1.43) **	1.24 (1.13,1.37) **
1 to 3 times a week		1.26 (1.15,1.38) **	1.35 (1.24,1.47) **	1.00 (0.91,1.09)
Father’s time spent on electronic screens on weekends (ref: Less than 2 h a day)				
2 or more hours per day		1.11 (1.02,1.19) *	0.50 (0.47,0.54) **	1.17 (1.08,1.26) **
Mother’s time spent on electronic screens on weekends (ref: Less than 2 h a day)				
2 or more hours per day		1.01 (0.93,1.09)	0.47 (0.44,0.51) **	1.13 (1.04,1.22) **
Whether the surrounding environment meets sports activities (ref: Something was lacking)				
Enough		1.42 (1.31,1.53) **	1.23 (1.14,1.33) **	1.01 (0.94,1.09)
Surrounding factors that hinder going out (ref: A few)				
Almost none		1.16 (1.07,1.25) **	1.13 (1.05,1.22) **	1.14 (1.05,1.22) *

Notes: I: College for professional training, bachelor’s degree or above; II: High school, technical secondary school or technical school; III: Junior middle school or below. Logistic regression was conducted to test the association between predictors and meeting guidelines; all predictors were included in each model (* *p* < 0.05; ** *p* < 0.01).

**Table 4:** The OR values for the eight combinations of three health-related behaviors.

	MVPA	ST	SLP	MVPA and ST	MVPA and SLP	ST and SLP	MVPA, ST and SLP
Sex (ref: girl)							
Boy	1.08	0.93	0.99	0.91	0.95	1.00	0.92
Age	0.95	0.93	0.79 **	0.94	0.76 **	0.74 **	0.70 **
Height	1.00	0.99 *	0.97 **	0.99	0.98 **	0.97 **	0.98 **
Weight	1.00	1.00	0.96 **	0.99	0.96 **	0.96 **	0.95 **
Urban and rural (ref: rural)							
Urban	1.05	0.85	0.98	1.20 **	1.04	0.99	1.28 **
Left-behind (ref: Yes)							
No	1.15	1.42 **	0.83	1.51 **	1.11	1.18	1.34 *
Father’s Educational level (ref: III)							
I	1.10	0.76 *	0.74 *	1.14	1.18	0.96	1.62 **
II	1.10	0.91	0.81	0.98	1.00	0.94	1.04
Mother’s Educational level (ref: III)							
I	1.02	0.75 **	0.71 **	1.09	1.10	0.92	1.46 **
II	1.20	1.05	0.83	0.99	1.06	1.04	1.04
Income (total annual household income) (ref: Less than 40,000 yuan)							
Over 80,000 yuan	1.22	0.65 **	1.10	0.99	1.60 **	0.78 **	1.44 *
40,000–80,000 yuan	1.07	0.74 **	0.98	1.05	1.30 **	0.71 **	1.05
Children’s athletic ability evaluated by parents (ref: general)							
Pretty good	1.34 **	1.15	1.00	1.74 **	1.46 **	1.27 **	1.95 **
Sports equipment (ref: Less than 1Sports equipment)							
2 or more Sports equipment	1.60 **	1.03	1.11	2.02 **	1.93 **	1.17	2.33 **
Parents’ time with their children on weekends (ref: Less than 2 h a day)							
More than 4 h a day	2.04 **	0.62 **	1.55 **	1.44 **	2.87 **	1.04	2.06 **
2 to 4 h a day	1.74 **	0.67 **	1.14	1.20	2.07 **	0.95	1.59 **
Parents’ attitudes towards sports activities (ref: Negative)							
Positive	1.94 **	1.51 **	1.19	2.69 **	2.30 **	1.60 **	3.66 **
Neutral	1.70 **	1.38 **	1.09	1.80 **	1.82 **	1.34 **	2.51 **
Father’s obesity status (ref: Normal)							
Obesity	1.01	0.75 **	0.94	0.67 **	0.93	0.74 **	0.77 **
Mother’s obesity status (ref: Normal)							
Obesity	0.92	0.70 **	0.77	0.56 **	0.85	0.74 *	0.66 **
Father’s exercise frequency (ref: Less than 1 a week)							
3 or more times a week	1.38 **	1.29 *	0.96	1.97 **	1.56 **	1.50 **	2.54 **
1 to 3 times a week	1.38 **	1.35 **	1.05	1.79 **	1.19	1.16	1.63 **
Mother’s exercise frequency (ref: Less than 1 a week)							
3 or more times a week	1.84 **	1.77 **	1.29	2.32 **	2.33 **	1.66 **	3.15 **
1 to 3 times a week	1.38 **	1.60 **	1.04	1.76 **	1.39 **	1.34 **	1.94 **
Father’s time spent on electronic screens on weekends (ref: Less than 2 h a day)							
2 or more hours per day	1.10	0.53 **	1.06	0.48 **	1.15	0.59 **	0.61 **
Mother’s time spent on electronic screens on weekends (ref: Less than 2 h a day)							
2 or more hours per day	0.94	0.46 **	0.91	0.40 **	1.01	0.57 **	0.45 **
Whether the surrounding environment meets sports activities (ref: Something is lacking)							
Enough	1.45 **	1.23 *	0.95	1.79 **	1.38 **	1.26 *	1.82 **
Surrounding factors that hinder going out (ref: A few)							
Almost none	1.32 **	1.16	1.26 *	1.53 **	1.37 **	1.56 **	1.53 **

Notes: I: College for professional training, bachelor’s degree or above; II: High school, technical secondary school or technical school; III: Junior middle school or below. (* *p* < 0.05; ** *p* < 0.01).

## Data Availability

The data presented in this study are available on request from the corresponding author. The data are not publicly available due to privacy.

## Appendix A

**Table A1:** Specific index content and evaluation methods.

Indicators	Assessment
Father’s Educational level	Divided into five items: elementary school and below, junior high school, high school or technical secondary school, university or college, master degree and above
Mother’s Educational level	Divided into five items: elementary school and below, junior high school, high school or technical secondary school, university or college, master degree and above
Income (total annual household income)	Divided into ten items: 20,000 and below, 20,100 to 40,000, 40,100 to 60,000, 60,100 to 80,000, 80,100 to 100,000, 100,100 to 150,000,150,100 to 200,000, 200,100 to 300,000, 300,100 to 500,000, 500,100 and above
Children’s athletic ability evaluated by parents	Including the child’s overall exercise level, the speed of mastering skills, specifically to the level of strength, endurance, agility and other dimensions. A total of 3 questions, each question was divided into 1–5 points and five levels of evaluation
Sports equipment	Including football, basketball, badminton, skipping rope, a total of 10 common sporting goods
Parents’ time with their children on weekends	Including no time at all, 2 h or less, 2 to less than 4 h, 4 to less than 6 h, 6 to less than 8 h, 8 to less than 12 h, 12 h and above, a total of 7 items
Parents’ attitudes towards sports activities	Including whether to encourage children to go out for sports, whether to enroll children in sports interest classes, whether children were more important to learning than sports, etc. A total of 7 questions, each question was divided into 1–5 points and five levels of evaluation
Father’s obesity status	Subjectively ask whether the child’s parents were obese
Mother’s obesity status	Subjectively ask whether the child’s parents were obese
Father’s exercise frequency	Including more than 3 times a week, 1–3 times a week, 1–3 times a month, lower frequency, never exercise and not very clear, a total of 6 items
Mother’s exercise frequency	Including more than 3 times a week, 1–3 times a week, 1–3 times a month, lower frequency, never exercise and not very clear, a total of 6 items
Father’s time spent on electronic screens on weekends	Including 1 h or less, 1-less than 2 h, 3-less than 3 h, 3-less than 4 h, 4 h and more and unclear, a total of 6 items
Mother’s time spent on electronic screens on weekends	Including 1 h or less, 1-less than 2 h, 3-less than 3 h, 3-less than 4 h, 4 h and more and unclear, a total of 6 items
Whether the surrounding environment meets sports activities	Ask parents whether there were suitable playgrounds for children’s sports, whether the surrounding areas were safe, sanitary, green, and whether there were people of the same age playing together. A total of 5 questions, each question was divided into 1–5 points and five levels
Surrounding factors that hinder going out	Ask the parents if there were too many pets, lack of publicity, lack of equipment, and over-population. A total of 5 questions, each question was divided into 1–5 points and five levels

**Table A2:** The OR values for the 8 combinations of three health-related behaviors. (controlling the children’s sex, age, height and weight factors).

	MVPA	ST	SLP	MVPA and ST	MVPA and SLP	ST and SLP	MVPA, ST and SLP
Urban and rural (ref: rural)							
Urban	1.05	0.91	0.99	1.24 **	1.00	1.00	1.23 **
Left-behind (ref: Yes)							
No	1.28	1.67 **	0.85	2.16 **	1.22	1.27	1.35
Father’s Educational level (ref: III)							
I	1.05	0.85	0.81	1.20	1.13	0.96	1.52 **
II	0.95	0.89	0.75 *	1.05	0.97	0.92	0.92
Mother’s Educational level (ref: III)							
I	1.11	0.93	0.88	1.30 *	1.17	0.99	1.49 **
II	1.20	1.05	0.83	0.99	1.06	1.04	1.04
Income (total annual household income) (ref: Less than 40000 yuan)							
Over 80,000 yuan	1.18	0.81	1.17	1.20	1.55 **	0.81	1.36 *
40,000–80,000 yuan	1.00	0.72 **	0.92	1.08	1.14	0.68 **	0.97
Children’s athletic ability evaluated by parents (ref: general)							
Pretty good	1.26 **	1.07	0.92	1.67 **	1.35 **	1.12	1.75 **
Sports equipment (ref: Less than 1Sports equipment)							
2 or more Sports equipment	1.66 **	1.05	1.07	2.17 **	1.98 **	1.14	2.40 **
Parents’ time with their children on weekends (ref: Less than 2 h a day)							
More than 4 h a day	2.14 **	0.75 *	1.45 *	1.46 **	3.07 **	1.06	2.24 **
2 to 4 h a day	1.60 **	0.65 **	1.00	1.16	1.98 **	0.89	1.59 **
Parents’ attitudes towards sports activities (ref: Negative)							
Positive	2.02 **	1.67 **	1.12	2.94 **	2.38 **	1.74 **	3.77 **
Neutral	1.69 **	1.50 **	1.08	1.95 **	1.91 **	1.40 *	2.28 **
Father’s obesity status (ref: Normal)							
Obesity	0.80	0.65 **	0.83	0.55 **	0.80 **	0.64 **	0.68 **
Mother’s obesity status (ref: Normal)							
Obesity	0.83	0.68 **	0.71 **	0.55 **	0.82	0.69 *	0.60 **
Father’s exercise frequency (ref: Less than 1 a week)							
3 or more times a week	1.42 *	1.38 *	0.92	2.04 **	1.51 **	1.47 **	2.30 **
1 to 3 times a week	1.60 **	1.48 **	1.08	2.16 **	1.27 *	1.16 *	1.80 **
Mother’s exercise frequency (ref: Less than 1 a week)							
3 or more times a week	1.72 **	1.65 **	1.13	2.17 **	2.08 **	1.40 *	2.81 **
1 to 3 times a week	1.57 **	1.86 **	1.09	1.93 **	1.52 **	1.30 *	2.16 **
Father’s time spent on electronic screens on weekends (ref: Less than 2 h a day)							
2 or more hours per day	1.06	0.55 **	1.07	0.49 **	1.16	0.66 **	0.60 **
Mother’s time spent on electronic screens on weekends (ref: Less than 2 h a day)							
2 or more hours per day	0.95	0.46 **	0.96	0.39 **	1.06	0.60 **	0.47 **
Whether the surrounding environment meets sports activities (ref: Something is lacking)							
Enough	1.44 **	1.32 **	0.95	1.92 **	1.44 **	1.28 *	1.86 **
Surrounding factors that hinder going out (ref: A few)							
Almost none	1.33 **	1.24 *	1.27 *	1.66 **	1.40 **	1.67 **	1.61 **

Notes: I: College for professional training, bachelor’s degree or above; II: High school, technical secondary school or technical school; III: Junior middle school or below. (* *p* < 0.05; ** *p* < 0.01).

**Table A3:** The OR values for the 8 combinations of three health-related behaviors. (controlling the children’s sex, age and body mass index).

	MVPA	ST	SLP	MVPA and ST	MVPA and SLP	ST and SLP	MVPA, ST and SLP
Urban and rural (ref: rural)							
Urban	1.05	0.91	0.98	1.25 **	1.00	0.99	1.23 **
Left-behind (ref: Yes)							
No	1.28	1.67 **	0.83	2.16 **	1.22	1.25	1.34
Father’s Educational level (ref: III)							
I	1.06	0.84	0.78	1.20	1.12	0.94	1.52 **
II	0.94	0.89	0.74 *	1.05	0.97	0.91	0.92
Mother’s Educational level (ref: III)							
I	1.11	0.92	0.86	1.30 *	1.16	0.98	1.49 **
II	1.10	1.05	0.77	1.03	1.00	1.00	0.97
Income (total annual household income) (ref: Less than 40000 yuan)							
Over 80000 yuan	1.19	0.81	1.13	1.21	1.54 **	0.79	1.36 *
40,000–80000 yuan	1.01	0.71 **	0.91	1.08	1.14	0.67 **	0.98
Children’s athletic ability evaluated by parents (ref: general)							
Pretty good	1.26 **	1.07	0.92	1.67 **	1.34 **	1.11	1.75 **
Sports equipment (ref: Less than 1Sports equipment)							
2 or more Sports equipment	1.66 **	1.04	1.04	2.18 **	1.95 **	1.12	2.39 **
Parents’ time with their children on weekends (ref: Less than 2 h a day)							
More than 4 h a day	2.14 **	0.74 *	1.42 *	1.46 **	3.05 **	1.04	2.24 **
2 to 4 h a day	1.61 **	0.65 **	1.00	1.16	1.97 **	0.88	1.59 **
Parents’ attitudes towards sports activities (ref: Negative)							
Positive	2.02 **	1.67 **	1.12	2.94 **	2.38 **	1.73 **	3.75 **
Neutral	1.69 **	1.50 **	1.05	1.94 **	1.92 **	1.37 *	2.28 **
Father’s obesity status (ref: Normal)							
Obesity	0.80	0.64 **	0.82	0.56 **	0.79 *	0.64 **	0.68 **
Mother’s obesity status (ref: Normal)							
Obesity	0.83	0.68 **	0.71 *	0.56 **	0.81	0.69 *	0.60 **
Father’s exercise frequency (ref: Less than 1 a week)							
3 or more times a week	1.42 *	1.37 *	0.91	2.03 **	1.52 **	1.45 **	2.30 **
1 to 3 times a week	1.60 **	1.48 **	1.09	2.15 **	1.28 *	1.16	1.80 **
Mother’s exercise frequency (ref: Less than 1 a week)							
3 or more times a week	1.72 **	1.65 **	1.13	2.17 **	2.08 **	1.40 *	2.81 **
1 to 3 times a week	1.57 **	1.86 **	1.08	1.93 **	1.52 **	1.29 *	2.16 **
Father’s time spent on electronic screens on weekends (ref: Less than 2 h a day)							
2 or more hours per day	1.06	0.54 **	1.08	0.49 **	1.15	0.66 **	0.60 **
Mother’s time spent on electronic screens on weekends (ref: Less than 2 h a day)							
2 or more hours per day	0.94	0.46 **	0.97	0.40 **	1.06	0.60 **	0.47 **
Whether the surrounding environment meets sports activities (ref: Something is lacking)							
Enough	1.44 **	1.32 **	0.94	1.92 **	1.43 **	1.27 *	1.86 **
Surrounding factors that hinder going out (ref: A few)							
Almost none	1.33 **	1.24 *	1.27 *	1.67 **	1.40 **	1.67 **	1.62 **

Notes: I: College for professional training, bachelor’s degree or above; II: High school, technical secondary school or technical school; III: Junior middle school or below. (* *p* < 0.05; ** *p* < 0.01).
